# Dr. Geo. C. Crawford

**Published:** 1872-03

**Authors:** 


### DR. GEO. G. CRAWFORD.

Our esteemed friend, Dr. Geo. G. Crawford, of this city, is arranging his interests in other portions of the State preparatory to making a "medical pilgrimage" to the great medical centres of Europe, where he expects to remain several years. Young in age, and blessed with ample means, we doubt not he will return to us to add fresh laurels to his already long list of triumphs in medicine and surgery. We wish him success, and a safe return.

